# Conserved amino acid markers from past influenza pandemic strains

**DOI:** 10.1186/1471-2180-9-77

**Published:** 2009-04-22

**Authors:** Jonathan E Allen, Shea N Gardner, Elizabeth A Vitalis, Tom R Slezak

**Affiliations:** 1 Lawrence Livermore National Laboratory, Livermore, CA, 94551, USA

## Abstract

**Background:**

Finding the amino acid mutations that affect the severity of influenza infections remains an open and challenging problem. Of special interest is better understanding how current circulating influenza strains could evolve into a new pandemic strain. Influenza proteomes from distinct viral phenotype classes were searched for class specific amino acid mutations conserved in past pandemics, using reverse engineered linear classifiers.

**Results:**

Thirty-four amino acid markers associated with host specificity and high mortality rate were found. Some markers had little impact on distinguishing the functional classes by themselves, however in combination with other mutations they improved class prediction. Pairwise combinations of influenza genomes were checked for reassortment and mutation events needed to acquire the pandemic conserved markers. Evolutionary pathways involving H1N1 human and swine strains mixed with avian strains show the potential to acquire the pandemic markers with a double reassortment and one or two amino acid mutations.

**Conclusion:**

The small mutation combinations found at multiple protein positions associated with viral phenotype indicate that surveillance tools could monitor genetic variation beyond single point mutations to track influenza strains. Finding that certain strain combinations have the potential to acquire pandemic conserved markers through a limited number of reassortment and mutation events illustrates the potential for reassortment and mutation events to lead to new circulating influenza strains.

## Background

Influenza A has evolved toward host specific mechanisms of infection leading to genetic divergence between human and avian strains. Sequence divergence is so striking that single nucleotide counts are sufficient for classifying the host type for most influenza strains when analyzing whole segment or whole genome data [1]. A notable exception is the H5N1 avian strain that crosses the species barrier and can lead to deadly human infection. The H5 surface protein, hemagglutinin (HA), in some cases is able to recognize human cell receptors [2,3] along with mutations that allow the virus to better survive in the upper respiratory tract [4]. To date, however, there are relatively low numbers of human H5N1 infections compared to the more human persistent subtypes, which may be in part due to inefficient human to human transmission [5,6]. In this study the influenza viruses from the pandemics of 1918, 1957 and 1968 with elements of avian (or avian-like) strains mixed with genetic elements persistent in humans [7-9] are used to provide a historic map of enduring genetic features from past pandemics and their circulation in current human, avian and swine strains [10].

Whole influenza genomes were searched for genetic markers conserved in pandemic strains that are associated with two features of infection: host specificity and high mortality rate. For host specificity a search was designed to find amino acid mutations in human influenza strains that were not observed in avian strains. The approach for defining host specificity markers closely followed the work of [11] which predicted positions in the genome associated with human host specificity. Other recent work [12] looked more broadly for human markers beyond the pandemic conserved regions. Both of these studies examined amino acid point mutations using differing measures for functional significance. In this study a new approach was developed to look for combinations of mutations in the genome that identify host specific evolutionary pressures beyond single point mutations. New mutations were identified that exhibit a co-variation mutation pattern. Evaluating mutation combinations allowed for the analysis of genetic markers where single point mutations failed to distinguish high and low mortality rate strains. In total 34 host specific and high mortality rate pandemic conserved markers were found. The ultimate goal of our study was to examine how the 34 pandemic conserved markers might re-emerge in a future single strain. While marker re-emergence in a single strain does not predict pandemic potential, their presence could highlight unexpected evolutionary events in circulating strains that warrant closer scrutiny.

Influenza genomes not used in the marker estimation process were searched for the presence or absence of the markers. The human host specific markers were sought in the recent avian strains infecting human (H5N1, H9N2, H7N3 and H7N7), the high mortality rate associated markers were sought in the avian strains and both marker sets were sought in non-avian non-human strains (e.g. swine, cat and others). The high mortality rate markers appeared in a wide variety of avian strains but the recent avian to human strain crossovers lacked most of the human strain specific markers. Human persistent strains retained human specific markers (by definition) but lacked most of the high mortality rate markers. Swine strains fell in the middle, carrying both high mortality rate and host specificity markers but with no single strain containing all 34 markers. Using a maximum parsimony principle, likely evolutionary pathways for the re-emergence of the 34 markers in a single strain were considered with a computational experiment. The fewest evolutionary events through reassortment and mutation needed for a single influenza strain to acquire all 34 markers in the presence of a second strain were counted. Starting with a small number of sequenced H1N1 human and swine strains, a mix with avian strains were found to acquire the 34 pandemic markers through a combination of 4 or fewer segment reassortment and amino acid mutation events.

## Results and discussion

The genetic marker identification procedure uses a discriminative classifier (a linear support vector machine [13]) with cross validation to build two models, one for host specificity and one for high mortality rate strains. The discriminative classifier is a computational tool that is designed to classify an unknown sample as belonging to one of two classes. Here one classifier model is designed to classify the influenza host type, the second model is designed to classify the influenza mortality rate type. Each model takes as input the 11 influenza proteins aligned and concatenated and classifies the strain in the case of host specificity as being human or avian. For mortality rate, input strains are divided into high and low mortality rate strain classes. The purpose for building the classifier is to find the positions in the influenza genome that are important in the model for accurately classifying input strains, a problem commonly referred to as the feature selection problem [14]. Candidate markers are found by building new classifiers that take as input a small subset of the influenza proteome. The input sets that lead to classifiers that match the accuracy of the original classifier (which uses the entire proteome as input) highlight the amino acid markers that are important for class discrimination. An iterative procedure is used. For the initial step all single amino acid positions are found that separate the two classes (human/avian or high/low mortality rate). The iterative step n identifies the n sized (potentially non-contiguous sequence) combinations that separate the data such that each combination does not contain a smaller sized combination that separates the two classes equally well. This procedure yields a set of non-redundant mutation patterns that separate the two classes. The iterative procedure is important so that a candidate marker is only included as part of a distinguishing pattern when it adds to the classification accuracy. So for example if position 21 in the PB2 protein distinguishes avian and human strains, then position 21 would not be included as part of another set of features (say position 22 in the PB2 protein). Only markers that contribute significantly to classification accuracy are included in the final result. Details on selecting candidate functional markers are given in the Methods section.

### Host specificity markers

Sixteen positions in the influenza genome were found to be associated with human host specificity. The markers were found on the non-structural protein 1 (NS1), non-structural protein 2 (NS2), matrix protein 1 (MP1), nucleoprotein (NP), acidic protein (PA) and the basic polymerase 2 (PB2) protein. Each strain was assigned a genotype, which showed whether the human consensus amino acid variant was present at each of the 16 positions. Strains excluded from the marker estimation process, human infections of avian origin [15] and non-human non-avian strains, were checked for evidence of an enrichment of human specificity markers relative to the remaining avian strains. With one exception the human infections of avian origin showed a genotype that was distinct from the most common avian genotype background but the number of accumulated human markers was small.

**Figure 1:**
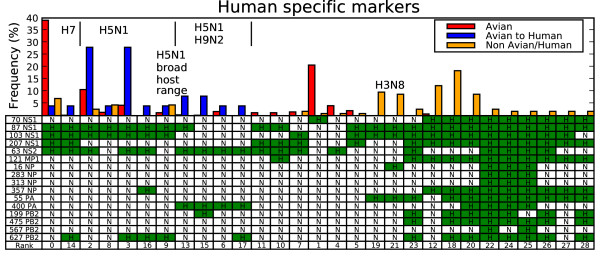
**Persistent human markers in non-human strains**. Each column in the table is a genotype with the bars showing genotype frequency for avian (red), avian to human crossovers (blue) and non-avian non-human strains (orange). A table entry with H (green) means the amino acid position has the human consensus for the amino acid position, and N means non-human consensus. The last row "Rank" labels each genotype and shows its frequency rank among avian strains. Rank is in increasing order with 0 being the most common genotype. Select strain subtypes are shown in the figure, with details given in the text.

Figure 1 shows the relative frequency of different host specificity genotypes among the sequenced samples with minimum 1% frequency for the three host categories: avian, human infections of avian origin and all other non-human non-avian host types. Redundant sequences that occur within the same region and year are collapsed to prevent over weighting heavily sequenced outbreaks. Columns in the table show each genotype configuration with the last row (Rank) reporting the rank of the genotype's relative frequency in avian strains. For example, the most common avian genotype is rank 0 and shown by the red bar to cover nearly 40% of all avian strains as well as a small percentage of other host infections shown by the blue and orange bars. Underneath the three frequency bars is the corresponding genotype: NHHHHNNNNNNNNNNN, which means that these strains have the human consensus marked 'H' at 4 protein positions: 87 NS1, 103 NS1, 207 NS1 and 63 NS2. The remaining 12 positions carry a non-human amino acid variant marked 'N'. Many of the human markers could be a consequence of persistent founder mutations from the ancestral 1918 pandemic strain, which gave rise to current circulating human strains. It is interesting to observe, however, that avian strains maintain each of the human consensus variants in the NS segment with species specific variation patterns. Twenty-four percent of the avian strains share the human consensus amino acid in position 87 NS1 spanning 35 distinct serotypes. Seventy-seven percent of the avian strains share at least one human consensus at one of the other positions in the NS segment, spanning 65 distinct serotypes. If the two sites evolved independently, 19% of the observed avian genotypes would be expected to share a human consensus at 87 NS1 and at least one of the other NS segment positions, however, only 2% of avian strains show this pattern. Half of these cases involve a collection of H3N2 avian strains that recently acquired the NS segment from a swine virus (Rank 12 in Figure 1). For position 70 and 87 in NS1, Lysine and Serine are the respective consensus amino acids in human. In avian strains, the combinations for the respective positions are Glutamic acid and Serine (58%), Lysine and Proline (26%), Glutamic acid and Proline (9%) and only rarely Lysine and Serine (2%).

The columns are grouped so that avian to human crossover genotypes are clustered into three groups labeled at the top of Figure 1 as: H7 (avian frequency rank 0 and 14), H5N1 beginning in 2003 (rank 2, 8, 3, 16 and 9) [7,16-19] and the H5N1/H9N2 Hong Kong outbreaks from 1997–1999 (rank 13, 15, 6, and 17) [20,21]. Additional similar genotype patterns are placed in adjacent columns. A pattern emerges from the two most common avian genotypes ranked 0 and 1 in Figure 1. These two genotypes cover 60% of the sequenced strains and span nearly all of the subtypes including H5N1, H9N2, H7N7 and H7N3. Among the lethal avian to human crossovers there are two genotypes that arise in humans that are not found in sequenced avian strains (rank 16 and 17). These cases could be examples of post infection mutations, or alternatively show the limits in the coverage of sequenced avian strains.

### High mortality rate markers

In a second experiment human influenza strains were separated into two groups: a high mortality rate group containing pandemic genomes selected from the 1918, 1957 and 1968 outbreaks, human H5N1 and the H1N1 1976 deadly New Jersey infection and a low mortality rate group containing all other whole genome human infection samples. As with the pandemic conserved host type markers, the high mortality rate markers were required to be positively identified in each of the sequenced strains associated with the three pandemic outbreaks (e.g. perfect conservation and no ambiguous sequence codes). Eighteen of 2,112 sequenced human influenza genomes (9 of 286 when samples were grouped by year, subtype and location) not in the high mortality rate class contained all 18 of the identified high mortality rate markers. These cases occurred in H2N2 and H3N2 strains from the 1960s and 1970s in years following their respective pandemics.

**Figure 2:**
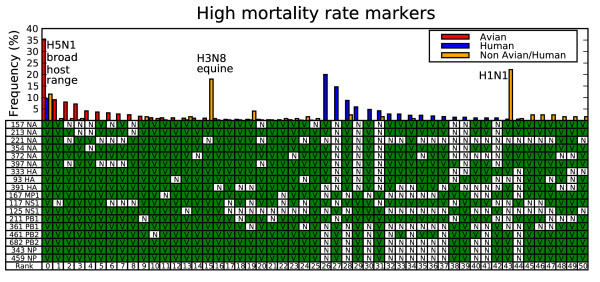
**High mortality rate genotypes**. Each genotype is specified by a column in the table, where the bars above the column reflect relative frequency in the sequenced genomes. V (green) means the genotype has the virulent consensus for the position, and N means non-virulent consensus. Bars above each table column mark the relative frequency for avian (red), human – both high mortality rate and low mortality rate cases (blue) and non-avian non-human strains (orange bars).

Figure 2 shows the high mortality rate genotypes among the sequenced samples with minimum 1% frequency for the three host categories. The figure shows that the human high mortality rate genotype is the most common avian genotype and that each avian strain has at least 13 of the 18 high mortality rate markers. Analogous to the co-variation pattern found in the NS segment for the human host type markers, the non-lethal human strains show that where the hemagglutinin (HA), neuraminidase (NA) subtype lacks the high mortality rate makers (rank 27, 29 and 31 in Figure 2) high mortality rate markers are found in other segments. The opposite also occurs (rank 26, 28 and 30 in Figure 2).

The most common non-human non-avian genotype (rank 43 in Figure 2) is a swine H1N1, which shares many of the high mortality rate variants but misses the mutations found on the NS and PB1 segments. The second most common subtype shares all but one of the high mortality rate variants and is circulating in horse (rank 15) but Figure 1 shows that H3N8 lacks most of the human host type markers (rank 19 and 21 in Figure 1). The complete high mortality rate variant (rank 0) are H5N1 cases that infect a broad host range including swine, tiger, domestic cat, civet, and stone marten. Figure 1 shows that these strains (most with the genotype with rank 9 in Figure 1) contain only a small number of human specific markers similar to the H5N1 human infections. The differences in genotypes show that swine host both strains found with human transmission markers or strains enriched with the high mortality rate markers. This could present an opportunity for two strains to mix and evolve into a swine strain with all 34 of the predicted pandemic conserved markers.

Recent work mixing avian H5N1 with human H3N2 in ferret models has shown that combining the H5N1 cell surface proteins with the internal human proteins need not lead directly to efficient ferret to ferret transmission, which serves as a model for human to human transmission [22]. In this approach only reassortment events were considered, highlighting the complexity that may be involved in acquiring the precise mix of genetic elements required for an H5N1 virus to acquire pandemic potential.

**Figure 3:**
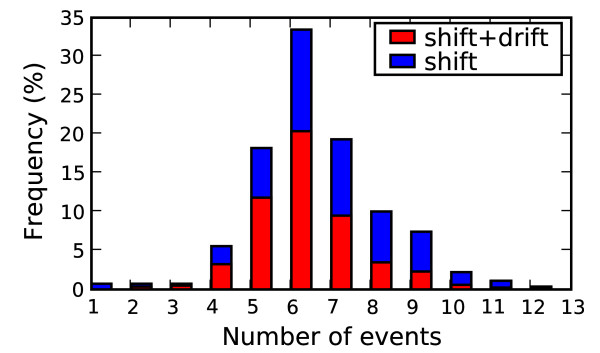
**Strain combinations with 34 markers**. Frequency distribution for the number evolutionary events needed to acquire the 34 pandemic markers. The 9 pairwise combinations are shown for human, avian and non-human non-avian. Red bar overlays show the average contribution of reassortment events (shift) to the total event count with mutations (drift).

To explore the steps needed to acquire the 34 genetic markers, hypothetical strain mixes were examined where pairs of genotypes sampled within one year difference were tested to simulate concurrent circulating strains. Two evolutionary events were considered: reassortment between segments counted as a single evolutionary event and an amino acid point mutation, also counted as a single evolutionary event. Each genotype was checked for the minimal number of events needed to acquire all 34 markers when mixed with a second strain. For completeness, all 9 pairwise combinations for the three host types were considered: human, avian and non-human non-avian. There were 269 distinct genotypes with 24,889 pairwise combinations and 187 distinct combinations of events that led to the 34 markers in a new strain. It is important to note that strain mixes that include a human strain already have the 16 human conserved markers and only lack the complement of high mortality rate conserved markers. Thus, human strains should require fewer mutation and reassortment events to acquire the 34 markers, compared to strain combinations between non-human influenza strains. Figure 3 shows the frequency distribution (in blue) for the fewest events needed for each of the 269 genotypes to acquire the 34 markers. The percentage of the blue bar covered by red is the relative contribution of reassortment events to the total. For example, in the case of 4 events, on average roughly half the events are attributed to reassortment. The histogram shows that on average the fewest events to acquire the 34 markers is almost always through a combination of reassortment and mutation. The figure points to two cases that are one mutation away from the 34 markers, a human H2N2 strain from 1968 and a H3N2 strain from 1971. These are examples of strains conserved from their respective recent pandemic ancestors and their presence (along with the other strains with the 34 markers previously referenced that persisted in the 1960s and 1970s) indicate that the 34 markers are not sufficient for causing pandemic potential.

Potentially novel strains with avian subtypes found to infect humans, which could circumvent existing human immunity (H7N7, H7N3, H7N2, H9N2, and H5N1), were examined more closely. Sixty-six distinct event combinations were found, but only a few cases required 4 events or less, which are summarized in Table 1. These potential paths involve 8 distinct genotypes from human and swine H1N1 strains, which acquire the two avian surface proteins plus one or two additional amino acid mutations on the NS1, PB1 or PB2 gene. Three of the 8 genotypes were observed in 2006 or later. The first sequenced strain from each location is given in Table 2. Although all of the human strains maintain all 16 human markers, they differ in the number of 18 high mortality rate markers present. Thus, different human strains require different numbers of mutations to acquire the 34 markers. For example, when starting with human H3N2 strains, 6 or more high mortality rate mutations are required in addition to the double reassortment with the HA and NA genes.

**Table 1:** Minimal evolutionary steps to acquire all 34 pandemic markers.

Initial strain	Region	Shift	Drift
H1N1 swine	Henan/Tianjin	H5, N1	199 PB2 117 NS1

H1N1 human	New Zealand	H9, N2	211 PB1
	Australia	H7, N2	117 NS1
	U.S.A., Asia	H5, N1	(one or both)

First column shows the initial strain, the second column shows region where strain is found, the third column shows double reassortments taken (Shift) and column four shows the mutations (Drift) taken. The human case (row 2) involves three subtypes (H9N2, H7N2, and H5N1) and one or two mutations.

**Table 2:** Strains sequenced since 2006 with 4 events or less needed to acquire the 34 markers.

Year	Location	Sample	Accession
2006	KENTUCKY	UR06-0010	157281296

2006	MICHIGAN	UR06-0015	157281277

2006	NEW YORK	8	118313168

2006	HENAN	01*	151335575

2006	TEXAS	UR06-0012	157281258

2007	CALIFORNIA	UR06-0435	157281639

2007	COLORADO	UR06-0111	157282703

2007	FLORIDA	UR06-0280	157282570

2007	ILLINOIS	UR006-018	157281334

2007	KANSAS	UR06-0140	157283026

2007	KENTUCKY	UR06-0028	157368127

2007	MISSISSIPPI	UR06-0048	157282646

2007	NEW YORK	UR06-0386	157281429

2007	OHIO	UR06-0100	157283121

2007	TEXAS	UR06-0025	157281620

2007	VERMONT	UR06-0050	157281467

2007	VIRGINIA	UR06-0109	157283102

The four columns are year sample was taken (Year), location of the sample (Location), the sample name (Sample) and GenBank accession (Accession). *H1N1 swine sample, all other samples are human H1N1 strains.

## Conclusion

A distinct genotype subset emerges from the avian background from which the human crossovers are derived with some strains adopting a limited number of human persistent markers. Overall, the human infections of avian origin have acquired no more than a few human specific markers, which suggests that avian strains are not rapidly acquiring human persistent markers through genetic drift. The high mortality rate markers are ubiquitous in the avian background and are distinct from the vast majority of human infections. While the host type markers clearly separate avian and human strains, there are a number of cases where descendants of the 1957 and 1968 pandemics continued to retain all of the predicted high mortality rate markers. Finding that classification accuracy for high mortality rate strains is lower than the host type classification weakens support for the notion of a single essential common set of high mortality rate markers. The reduced classification accuracy comes primarily from the fact that the H2N2 sequences continue to maintain the 18 markers into the 1960s, well past the associated pandemic. Thus, these 18 markers do not clearly distinguish between pandemic and non-pandemic associated H2N2 strains. Instead the results support the hypothesis that additional factors play an important role in determining the mortality rates of a specific strain. This highlights the potential importance to pandemic potential of host immunity and antigenic novelty. Even in the case of host type markers where classification accuracy is very high, markers could be missed. For example, the HA and NA genes play a critical role in host specific infection, but this study focused specifically on the persistent markers, and host specificity markers were found only on the more heavily conserved internal proteins. Additional potentially important host type markers that are not persistent should still exist.

It is worth noting that 5 of the 18 high mortality rate markers lie on the NA or PB1 segments implying that they were independently introduced into the three respective pandemic outbreaks [7]. Aside from the 18 high mortality rate markers persisting in H2N2 strains past the 1957 pandemic time frame, the markers give an overall high degree of classification accuracy and, therefore, a potentially useful common, although not sufficient, set of associated genetic factors. Among the high mortality rate strains not associated with a pandemic, only the 1976 H1N1 isolate lacks all 18 markers (4 are not present). Because the 1976 sample is a small contributor to the total number of high mortality rate features, it does not significantly contribute to the classification model. Substituting a single alternate 1976 swine strain for example, would have limited impact on the markers chosen unless more strains were added or a single strain was given the same weight as the pandemic strains in which perfect conservation is required. In this case mixing low mortality rate strains into the high mortality rate class would substantially alter the reported set of persistent markers. Requiring perfect conservation with the 1976 H1N1 strain would reduce the number of candidate markers to 14 (or less if an alternate 1976 swine strain were used). Similarly, swapping in nearly any other H3N2 sequence from the low mortality rate class, including those from the 1970s would alter the candidate marker set due to a lack of conservation.

Evolutionary pathways through reassortment and mutation show that strain combinations starting with H1N1 human and swine need the fewest events to acquire the pandemic conserved markers. Several of these pathways would lead to novel strains with H5N1 subtypes that could challenge human immunity. The potential need for an extended time or number of exposures for strains to acquire the human persistent mutations combined with the high mortality rate markers associated with avian strains suggests how swine could act as a mixing vessel where both human specific and high mortality rate markers are found to persist. Additional work may reveal restrictions that limit the strain combinations that lead to viable new strains. Measuring the rate of co-infection in swine and human, particularly in cases where an avian like strain is suspected to be present, could provide additional data for more precisely modeling the likelihood of the reassortment events that combine with mutations to facilitate mutation combinations important to infection.

## Methods

A pattern classification approach [23] is used with heuristic feature selection [14,24] to predict the candidate markers. Taken as input is a multiple sequence alignment (using MUSCLE [25]) for a collection of influenza genomes, where the 11 proteins are concatenated together. Each position in the alignment is converted to a bit vector of length 21, where an entry of 1 in the vector indicates the presence of one of the 20 amino acids or an insertion symbol. For an input alignment of length *x *(and 21 × *x *length bit vector), to find all *n *sized mutation subsets, *x *choose *n *combinations are checked, which is time prohibitive even for small *n *when *x *is large. A heuristic is used to exploit the information obtained from the linear support vector machine (LSVM) to reduce the size of *x *to 60 and limit *n *to 10. Note that even this size (~7 × 10^10^) in theory could be too large to efficiently process. Since smaller combination sizes were found, the search space size was sufficiently reduced to compute a solution. The LSVM computes weights for each position in the alignment reflecting the relative influence on the classifier. These weights are used to select the *x *most heavily weighted mutations from which to consider combinations. A similar approach was used in document classification [26] and a related approach was taken to classify 70 antibody light chain proteins [27]. LSVM code was developed by modifying the software package LIBSVM [28].

The expected classification accuracy is defined by the accuracy of the LSVM using the aligned proteome as input and 5-fold cross validation. Similar to the approach taken by [11] for human specific markers, sequences in the multiple sequence alignment used for training the classifier were labeled either human or avian depending on the host, excluding the avian to human crossover samples (H5N1, H9N2, H7N7 and H7N3) from training and testing. The 2,026 human persistent strains and 1,018 avian strains were grouped by time, location and subtype, with representative samples chosen at random to yield 281 distinct human strains and 560 distinct avian strains. Classifier accuracy was estimated by randomly dividing the data set into 5 non-overlapping partitions. The classifier was trained on 4 of the partitions and accuracy was measured by the percentage of correct classifications on the fifth partition, with the percentage of correct classifications calculated separately for each class to account for the difference in class size. The average of all 5 tested non-overlapping partitions was calculated giving two accuracy values (one for each class) and the final accuracy measure was the average of these two values. The 34 pandemic conserved markers given in this report were required to be positively identified in every sequenced strain in each of the three pandemic outbreaks without deviation from the majority consensus. This led to three markers reported in [11] that were excluded from this report for lack of conservation or positive identification (when an ambiguous sequence code was present) in one of the sequenced strains associated with the pandemic outbreaks.

The host specificity classifier misclassified 2 human and 2 avian strains for a classification accuracy of 99.5%. The classification errors appeared to be due to recent reassortment events that suggest the presence of influenza genomes that are a mix of both human and avian strains [29].

The high mortality rate data set was constructed using the same procedure as the host type dataset and the same 5-fold cross validation procedure was used to estimate accuracy. A total of 111 influenza genomes were classified as high-mortality rate strains and 2,001 were classified as low-mortality rate strains, with a non-redundant subset taken for training (35 high mortality rate, and 255 low mortality rate). The percentage of high and low mortality rate strains that were correctly classified was 96.2% and 96.9% respectively (an average of 96.6%). The lower accuracy for the high mortality rate classifier compared to the host type classifier likely highlights the genetic complexity associated with high mortality rate and the influence of other important factors such as host interaction.

Newly generated classifiers using only a small subset of the aligned proteomes as input were required to match the original classifier accuracy (99.5% for host type and 96.6% for high mortality rate type) within a margin of error defined by a confidence threshold. The confidence thresholds were defined by confidence intervals assuming 1 sided t-test comparisons using the standard deviation in the cross validation tests. Lowering the classification accuracy threshold allowed for the possibility of undetected reassortment events and other potential strain labeling errors (such as host interaction factors) that preclude perfect separation of class types.

The genotype analysis shown in Figures 1 and 2 includes 193 non-human non-avian influenza strains. All data was downloaded from the NCBI influenza whole genome database [30].

### Finding markers tied to function

**Figure 4:**
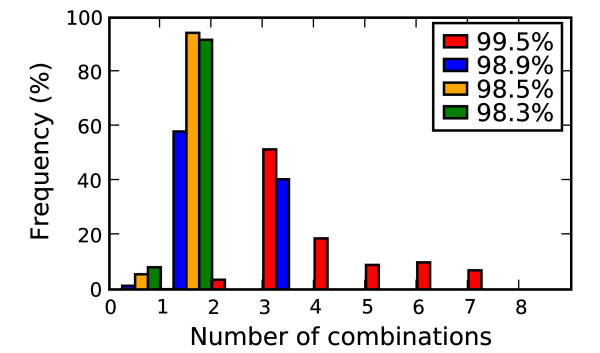
**Mutation combination sizes**. Relative frequency of mutation combination sizes for different classification accuracy thresholds. Red is the highest accuracy cut off, followed by blue, orange and green.

Figure 4 shows the frequency distribution for the size of amino acid combinations (combinations up to size 10 were checked) that distinguish avian and human strains at the different accuracy thresholds. The highest accuracy threshold of 99.5% (red bar in Figure 4) requires using more mutations per combination to accurately discriminate host type. For example, a minimum of 3 amino acid positions are required, with most combinations using 4 or more amino acid positions. By contrast, at the lowest accuracy thresholds, only single or pairs of amino acids are needed.

**Figure 5:**
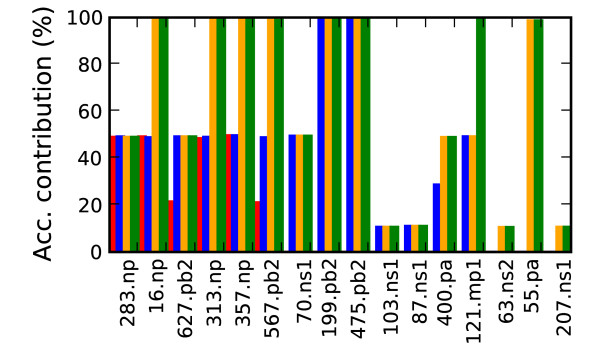
**Host marker classification accuracy**. Relative contribution of the human transmission markers to classification accuracy (Acc. = Accuracy). Positions increasing classification accuracy by at least 10% are shown. The colored bars show each mutation's contribution at the 4 different accuracy thresholds. Red is the highest accuracy cut off (99.5%), followed by blue (98.9%), orange (98.5%) and green (98.3%).

In Chen et al. (2006) functional significance was calibrated to detect the 627 PB2 mutation. A feature of the 627 PB2 mutation is that the human variant (Lysine) was found in 1% of the background avian flu and 23% of the H5N1 avian flu (~5% total) suggesting less human specific selective pressure. Thus distinguishing at the minimal accuracy threshold (set at 98.3%) using 627 PB2 required at least one additional marker. From the combinations of amino acid positions used for discrimination, an individual marker's functional significance was determined by two criteria. The marker must be part of a combination of mutations that separates the two phenotype classes with the same degree of accuracy (at one of the four confidence thresholds) that was achieved using the complete proteome alignment as input. Second the marker's individual contribution to the combination's classification accuracy must be above a minimal threshold defined by the distribution of observed contribution values. A mutation's contribution value was measured by the maximal increase in classification accuracy gained by adding the marker as a feature to one of the classifiers that met the minimal accuracy requirements. For example, mutation 627 PB2 could be combined with several additional mutations to make an accurate classifier. The classification accuracy of each of the additional mutations was measured without including 627 PB2 and compared to the accuracy when including 627 PB2, with the maximal difference being 627 PB2's contribution value. Figure 5 plots the contribution values for each candidate marker's maximal contribution to classification accuracy for the 4 different accuracy thresholds. At one end of the spectrum are markers like position 199 PB2 which is shown in Figure 5 to accurately classify close to 99% of the samples, without looking at any other positions in the proteome. Most positions add little to the host type discrimination, with accuracy contributions well below 1% (for clarity these positions were excluded from Figure 5). The figure shows the 16 mutations that stand out by their contribution of at least a 10% increase in accuracy at one of the four accuracy thresholds.

Ten of the 13 pandemic conserved host specificity positions reported in [11] were found. The 3 remaining markers (702 PB2, 28 PA and 552 PA) were not predicted due to lack of conservation among the pandemic strains. The host specific mutations reported here but not in [11] are attributed to the use of mutation combinations to guide the search for new genetic markers. Two mutations of note not reported by [11] that gave at least a 5% increase in accuracy at the highest classification accuracy threshold (99.5%) were 400 PA and 70 NS1. The 400 PA human consensus amino acid was Leucine and 3% of the avian strains had Leucine, with the remainder split between Serine and Proline. In the case of 70 NS1, 99.6% of human samples had Lysine along with 23% of the avian strains. (The avian consensus amino acid was Glutamic acid.)

**Figure 6:**
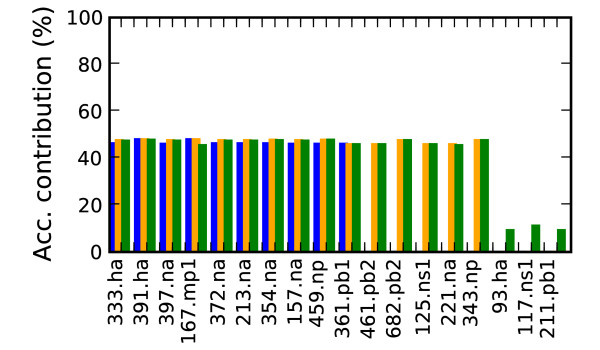
**High mortality rate marker classification accuracy**. Contribution to classification accuracy of high mortality rate markers (Acc. = Accuracy). Positions increasing classification accuracy by at least 5% are shown. Blue is the highest accuracy cut off (94.8%), followed by orange (93.5%) and green (92.8%).

Figure 6 shows the analysis for finding the high mortality rate type mutations. No single mutation contributed more than 50% to the classification accuracy, which illustrates the complexity of high mortality rate classification. Multiple mutations were required, but even considering combinations of size less than 10 precluded classification accuracy levels that matched the initial classifier accuracy using the whole genome as input. The marker combinations were found to reach the accuracy levels only at the 3 lower thresholds of 94.8%, 93.5% and 92.8% but not at the highest threshold of 96.6%

## Authors' contributions

JEA, SNG and TRS conceived and designed experiments. JEA implemented experiments and drafted the manuscript. JEA, SNG, EAV and TRS analyzed results and edited the manuscript.
